# Revealing Personality Triggers for Media Vicarious Traumatization: A Fuzzy-Set Qualitative Comparative Analysis

**DOI:** 10.3390/healthcare10101850

**Published:** 2022-09-23

**Authors:** Xin Huang, Yibin Shi, Guannan Gao

**Affiliations:** 1 School of Journalism and Communication, Wuhan University, Wuhan 430072, China; 2 School of Arts, Hubei University of Education, Wuhan 430205, China

**Keywords:** personality traits, media vicarious traumatization, air crash, university students, fsQCA

## Abstract

People may experience media vicarious traumatization due to frequent exposure to media coverage of disasters. Currently, the influential relationship between personality traits and media vicarious traumatization still lacks systematic and in-depth research. Based on the MU5735 airplane crash, this study explored the effects of configurations of personality traits on media vicarious traumatization by analyzing data from 331 Chinese university students (Mage = 22.63 years, SD = 2.67, range = 18 to 29, n = 186 male and n = 145 female) using Fuzzy-set Qualitative Comparative Analysis (fsQCA). The results revealed that five combinations of the Big Five personality traits could lead to media vicarious traumatization, the combinations of configurations are: (1) high conscientiousness, high agreeableness, and high neuroticism; (2) high conscientiousness, high extraversion, and high agreeableness; (3) high extraversion, high neuroticism, low conscientiousness, and low agreeableness; (4) high openness, high extraversion, high agreeableness, and high neuroticism; (5) high extraversion, high agreeableness, low openness, and low neuroticism. Furthermore, sociodemographic variables (gender, age, and education) interacted with personality traits and also resulted in different configurations of media vicarious traumatization. This study indicates the asymmetric relationships between personality traits and media vicarious traumatization, identifies the vulnerable groups to facilitate targeted trauma interventions for university students according to different configurations, and provides a reference for public psychological relief efforts in emergencies.

## 1. Introduction

On 21 March 2022, a Boeing 737 (MU5735) of China Eastern Airlines crashed in Teng County, Wuzhou, Guangxi Zhuang Autonomous Region, killing all 123 passengers and 9 crew members [1]. Journalists rushed to the site after the crash and reported the latest situation and the progress of rescue efforts through mass media and social media. People witnessed the tragic scenes through the ongoing media coverage. Studies have indicated that frequent exposure to all-round and continuous media coverage of a recent disaster negatively affects viewers’ mental health [2,3] and may trigger vicarious traumatization [4]. Vicarious traumatization initially referred to the phenomenon that psychotherapists experience adverse psychological reactions due to being unintentionally influenced by the bidirectional interaction between consultation and interview through long-term contact with trauma victims or the mentally ill [5]. Vicarious traumatization is a psychological disorder caused by indirect exposure to traumatic events [6]. Nowadays, the vicarious traumatization concept has been used in the research field of disasters, referring to the individual psychological abnormalities indirectly resulting from being harmed by what is beyond psychological and emotional endurance after witnessing numerous brutal and devastating disaster scenes [7]. A previous study found that psychological abnormalities stemming from sympathy for trauma survivors could lead to severe physical and mental suffering and even mental breakdown [8]. Holman et al. [9] indicated that people with six or more hours of daily media exposure in the week following the Boston Marathon bombings (BMB) had a ninefold increased likelihood of reporting high acute stress compared with those directly exposed to BMB. Therefore, some psychologists have called for vigilance against vicarious trauma induced by related coverage following this air crash [10].

### 1.1. Media Exposure and Media Vicarious Traumatization

People can be exposed to vivid disaster coverage anytime and anywhere through various media channels and vicariously experience disasters, as media technology has deeply penetrated social life [3]. Media serves as an essential trigger for vicarious traumatization [11]. In line with this, Blanchard et al. [4] noted that the related coverage (information on the attacks and reparative actions such as donating blood, attending vigils, etc.) of the attacks of 11 September 2001 in America could cause vicarious traumatization in university students. Turnbull et al. [12] also stated that the audience might experience vicarious traumatization symptoms, such as depression and anxiety, when exposed to audio and images of adverse events shared on social media. In addition, Zhong et al. [13] found that as users consistently got information about COVID-19 on social media during the pandemic, their vicarious traumatization levels elevated. Additionally, according to Liu et al.’s [14] study, COVID-19-related information released on official media, commercial media, social media, and overseas media caused varying influences on people’s vicarious traumatization levels. It is worth noting that fake information and conspiracy beliefs are highly disseminated in such overwhelming traumas, which may increase mental distress and the possibility of vicarious trauma [15]. Studies above all posited that media exposure significantly relates to vicarious traumatization. Further, vicarious traumatization induced by media exposure was named media vicarious traumatization in a recent study [15].

### 1.2. Personality Traits and Media Vicarious Traumatization

According to Lerias and Byrne’s [16] study, vicarious traumatization varied from person to person, and not everyone indirectly exposed to a traumatic event would experience it. Furthermore, differences in individual characteristics affect the likelihood that people will experience vicarious traumatization. Variances of individual characteristics in thoughts, senses, and behavior are called personality traits [17], reflecting a personal tendency to respond in a particular way under certain circumstances [18]. Previous studies have found that personality traits are essential predictor variables of vicarious traumatization [19,20,21]. The Big Five personality traits are commonly viewed as critical characteristics forming the fundamental of personal differences [22], including five dimensions of openness, conscientiousness, extraversion, agreeableness, and neuroticism [23]. Openness is the propensity to favor a wide variety of novel experiences. Conscientiousness is the quality of self-discipline and responsibility. Extraversion refers to the tendency to be outgoing and vibrant. Agreeableness is the propensity to be compassionate and eager to cooperate with others. Lastly, neuroticism describes the tendency to feel unpleasant emotions such as anxiety, anger, and depression. The Big Five personality traits have been applied in vicarious traumatization studies. For example, Bakhshi et al. [20] found that people with significant neuroticism were more susceptible to high levels of vicarious traumatization. Maguire and Byrne [21] revealed that agreeableness was a personality trait with vulnerability. Mǎirean and Turliuc [24] highlighted the negative correlation between extraversion and vicarious traumatization as well as that between conscientiousness and vicarious traumatization.

Past studies have also supported the relationship between personality traits and media vicarious traumatization. Sun [25] identified that the trauma caused by the media in a disaster event was related to the patterns of individuals’ media information processing; the more one was motivated, engaged, and effortful in following the disaster coverage, the more likely one would be stricken or influenced by the disaster event. Moreover, such behavior is significantly influenced by personality traits [26]. According to the cognitive-experiential self-theory, humans operate by two fundamental information-processing systems: a rational system and an experiential system. The two systems relate to personality traits [27]. Specifically, both Pacini and Epstein’s [28] and Curtis’ studies [29] showed that people high on conscientiousness and openness tended to process information logically and rationally and believed they were self-controlled and immune to being affected by media content. Consequently, it can be inferred that such persons may not be vulnerable to media vicarious traumatization. In contrast, people high on extraversion, agreeableness, and neuroticism prefer to process information with empirical [28] and emotional orientations [29], and thus are easily influenced by media content. So accordingly, these people are presumed to be at greater risk of suffering media vicarious traumatization. As a result, we can indirectly speculate that the influential relationship exists between personality traits and media vicarious traumatization. However, direct academic research on this relationship is still lacking.

### 1.3. Sociodemographic Variables and Media Vicarious Traumatization

In addition to personality traits, sociodemographic variables also influence the likelihood that an individual will experience media vicarious traumatization.

Gender differences have been found in many studies on victims of vicarious trauma. Blanchard et al. [4] found that women experienced more severe depressive symptoms and stress reactions after exposure to disaster reports compared to men. In a study of forest fires in Australia, Byrne et al. [30] found that after learning about the fire event on television or radio, 73.9% of women had symptoms of vicarious trauma, compared to only 26.1% of men. Furthermore, Ghahramanlou and Brodbeck [31] noted that younger age was a risk factor for vicarious trauma. Studies have shown that vicarious traumatization is often higher in younger therapists when they are exposed to traumatic material from their clients [32,33]. Similarly, educational attainment is also considered a variable leading to media vicarious traumatization. Researchers have also found that more educated people are less prone to post-traumatic stress disorder symptoms [34], and less educated people show severe vicarious trauma [35].

### 1.4. Research Questions

To fill the current research gap, we intend to study the relationship between personality traits and media vicarious traumatization. Notably, previous scholars mostly explored the effects of personality traits by discussing the net effect of each trait, which has certain limitations. In fact, each person is comprised of various personality traits, and no single trait can function on its own [36]. For this reason, we think it is more realistic to conduct this study from the perspective of factor configuration [37]. Therefore, this study proposes the following question:

Question 1: What configurations of personality traits are more likely to lead to media vicarious traumatization?

Question 2: Do sociodemographic variables interact with personality traits and are more likely to lead to media vicarious traumatization?

This study makes three contributions. First, it advances the studies on the influencing factors of media vicarious traumatization. Second, it contributes to a deeper comprehension of the influential relationship between personality traits and media vicarious traumatization. Third, it provides references for preventing and treating media vicarious traumatization.

## 2. Materials and Methods

### 2.1. Data Collection

Given that university students are susceptible to information on social media [38,39] and prone to experience media vicarious traumatization [4], this study took them as the sample.

The data collection was from 23 March to 10 April 2022, consigned to the Wenjuanxing platform, an online survey agency with a sample library of over 300 million members from all backgrounds. All respondents were told of the objective of this research and the anonymity of their participation before filling out the questionnaire. In addition, we set a filter question before the questionnaire: Have you followed coverage and information about the crash of China Eastern Airlines MU5735? Only those who selected ‘Yes’ would be able to answer the questionnaire; if they selected ‘No’, the survey would be automatically closed. We recycled 382 questionnaires in this way and ultimately obtained 331 valid samples after eliminating any incomplete ones.

### 2.2. Measures

This questionnaire consisted of two parts: the first was measures of basic personal information, including gender, education, and age; the second measured variables of personality traits and media vicarious traumatization.

#### 2.2.1. Personality Traits

Personality traits were measured using the Big Five Inventory-Short Version (BFI-S) developed by Gerlitz and Schupp [40], which is an indirect, reliable, and widely used scale of personality traits [41,42]. BFI-S has 15 items, with every 3 items measuring one personality trait: openness (e.g., “I see myself as someone who is original, comes up with new ideas”), conscientiousness (e.g., “I see myself as someone who does a thorough job”), extraversion (e.g., “I see myself as someone who is talkative”), agreeableness (e.g., “I see myself as someone who is sometimes rude to others”), and neuroticism (e.g., “I see myself as someone who worries a lot”). Like many sociological studies [41,43], this study used a concise scale to lessen the respondent burden. The responses were rated on a 5-point scale (1 = strongly disagree; 5 = strongly agree). The score of this scale ranged from 15 to 75. Cronbach’s α for the scale in this study ranged from 0.72 to 0.79 (openness 0.79, conscientiousness 0.72, extraversion 0.76, agreeableness 0.76, neuroticism 0.74).

#### 2.2.2. Media Vicarious Traumatization

Media vicarious traumatization was measured with Media Vicarious Traumatization Scale used in Liu et al.’s [14] study, which was used to assess the vicarious trauma experienced by people exposed to media during the COVID-19 pandemic. It comprised seven items, such as “I was exposed to distressing news and experiences via media” and “It is hard to stay positive and optimistic given some of the information I get from the media”. The responses were rated on a 5-point scale (1 = strongly disagree; 5 = strongly agree). The score of this scale ranged from 7 to 35. A higher score indicates a higher level of distress. Cronbach’s α of this scale in this study was 0.87.

Items for all these variables were rated on a 5-point Likert scale (1 = “strongly disagree” and 5 = “strongly agree”). To ensure the accuracy and comprehensibility of this questionnaire, we first invited a translator to convert these items into Chinese since the original scale was in English, and then we reviewed the text with the help of a communication specialist and a psychologist. Moreover, this questionnaire was piloted on 20 university students (undergraduate and graduate) to improve the validity. According to the results of this pilot study, Cronbach’s α for all variables were above the threshold of 0.7. Despite this, it is necessary to clarify that our ultimate analysis did not incorporate the data obtained in this pilot study.

### 2.3. Statistical Analysis

Statistical analysis was carried out using Fuzzy-set Qualitative Comparative Analysis (fsQCA). fsQCA is a technique combining qualitative and quantitative approaches, widely used to study complex configurations of structures [44]. Unlike traditional analysis methods, fsQCA can analyze complex asymmetric relationships between the combinations of antecedents and the outcome [45,46]. Consequently, the premise of using fsQCA is that the outcome depends on at least one configuration of causal conditions instead of on a single factor and its net effect [45]. Researchers have suggested applying the analysis method of asymmetric configuration in studies on complex phenomena, particularly those on human behavior unlikely to follow a symmetrical stance [47,48].

Individual reactions and behavior are shaped by the interaction of various personality traits rather than by the function of a single one [36,49]. As a consequence, to fully explain multiple configurations of personality traits that influence a person’s media vicarious traumatization, we used fsQCA to investigate the causality between personality traits and media vicarious traumatization, analyze the complex interactions among these personality traits, and further discuss the asymmetric relationships between these combinations of personality traits and media vicarious traumatization.

This study analyzed data using the fsQCA software 3.0.

## 3. Results

### 3.1. Sample Characteristics

Table 1 shows the sociodemographic characteristics of participants. Of all these participants, males accounted for 56.2% (n = 186) while females were 43.8% (n = 145); undergraduate students were 79.8% (n = 264) and graduate students were 20.2% (n = 67); participants aged 18–24 reached 72.8% (n = 241) and those aged 25–29 were 27.2% (n = 90).

### 3.2. Common Method Bias

Before proceeding to the fsQCA procedure, we checked the measurement properties of the variables. First, the common method bias was assessed using Harman’s single factor test [50]. According to the results, the strongest factor accounted for merely 22.62% of the total variance and was much lower than the conservative threshold of 40% [51]. Subsequently, we performed a full collinearity test to obtain the full collinearity variance inflation factors (VIF) for all constructs, revealing that all VIF values were below the threshold of 3.3 [52]. So accordingly, common method bias does not exist in our research.

### 3.3. Reliability and Validity Assessment

To assess the reliability and validity of the BFI-S and the Media Vicarious Traumatization Scale, this study employed Confirmatory Factor Analysis (CFA) to ascertain the measurement model. Table 2 and Table 3 present the results from our analysis of the measurement model.

First, reliability was assessed with Cronbach’s α. Table 2 shows the final results that Cronbach’s α values for all variables range from 0.72 to 0.87 and are above the threshold of 0.7 [53], showing satisfactory reliability of the measures used in this study.

Second, we evaluated convergent validity with factor loading, composite reliability (CR), and the average variance extracted (AVE), and evaluated discriminant validity with the heterotrait-monotrait (HTMT) ratio. Table 2 shows that factor loading values are from 0.61 to 0.89, CR values are from 0.84 to 0.91, and AVE values are from 0.61 to 0.70, which are, respectively, above the thresholds of 0.5, 0.7, and 0.5 [54]. As shown in Table 3, the HTMT ratios for each construct of the BFI-S range from 0.01 to 0.62, which are lower than the threshold of 0.85 [55]. The results demonstrate satisfactory convergent validity and discriminant validity of the measures used in this study.

**Table 3:** Discriminant validity measured by HTMT (the Big Five Inventory-Short Version). (The HTMT is the mean value of the indicator correlations across constructs relative to the mean of the average correlations for the indicators measuring the same construct [56]).

	1	2	3	4	5
1. Agreeableness					
2. Conscientiousness	0.62				
3. Extraversion	0.37	0.48			
4. Neuroticism	0.11	0.05	0.25		
5. Openness	0.29	0.34	0.31	0.10	

### 3.4. Qualitative Comparative Analysis

#### 3.4.1. Data Calibration

To use fsQCA, all variables need to be calibrated into fuzzy sets ranging from 0 (full non-membership) to 1 (full membership) [57]. In this study, fuzzy sets express personality traits, of which the fuzzy value of 1 means that someone is extremely high on a particular personality trait, while 0 has the opposite meaning. Before the calibration, three meaningful thresholds need to be chosen as the basis [57], defining the degree of membership in the fuzzy set for each personality trait. Based on the data we obtained and referred to the practice of previous research [57,58], this study adopted the direct calibration method and chose the values 0.95, 0.05, and 0.50 as the qualitative thresholds, respectively corresponding to full membership, full non-membership, and cross-over point. fsQCA can be applied to crisp (i.e., binary) sets, fuzzy sets, and mixtures of fuzzy and crisp sets [57]. Thus, gender (Male = 0, Female = 1), age (18–24 = 0, 25–29 = 1), and education (Undergraduate student = 0, Graduate student = 1) were treated as crisp sets in this study.

#### 3.4.2. Single-Factor Necessity Analysis

The necessity analysis is recommended to identify the necessity of each causal condition. A causal condition is necessary if it has a consistency value higher than 0.9 [57]. Table 4 shows the results of the necessary analysis, and the highest consistency is 0.758. Thus, the conditions necessary to cause media vicarious traumatization do not exist.

#### 3.4.3. Sufficiency Analysis of Conditional Configuration

Sufficiency analysis is mainly based on the truth table that contains all possible configurations (or combination paths). The truth table (Table 5) of this research consisted of 32 ($2^{5}$) different configurations since there were five antecedent conditions (openness, conscientiousness, extraversion, agreeableness, and neuroticism). We first sorted the truth table by frequency and consistency and set a frequency threshold to simplify the configurations of the initial truth table. As the number of our sample was more than 150, the threshold was set at 3 [59]. Moreover, we set raw consistency at 0.80 [57] and proportional reduction inconsistency (PRI) consistency at 0.60 [60]. Finally, three solutions were obtained through the Quine-McCluskey algorithm, namely complex solution, parsimonious solution, intermediate solution. The intermediate solution was chosen for result interpretation because it includes only simplifying assumptions and yields greater interpretability [57].

Table 6 reveals five configurations of personality traits related to media vicarious traumatization. It shows that the consistency of each solution and the overall solution are all above the threshold of 0.75. Further, the overall solution coverage of 0.683 implies the high explanatory power of personality traits.

To strengthen our understanding of media vicarious traumatization, we explored how three primary characteristics (i.e., gender, age, and education) of university students lead to media vicarious traumatization under the combination with the five personality traits. Table 7 shows the final configuration results.

The nine configurations are: (1) ~Conscientiousness * Extraversion * ~Agreeableness * Neuroticism * Gender * ~Age * ~Education; (2) Conscientiousness * Extraversion * Agreeableness * Neuroticism * Gender * ~Age * ~Education; (3) Openness * Extraversion * ~Agreeableness * Gender * ~Age * ~Education; (4) Openness * Conscientiousness * Extraversion * Agreeableness * ~Neuroticism * Gender * ~Age; (5) ~Openness * Conscientiousness * ~Extraversion * Agreeableness * Neuroticism * Gender * Age * ~Education; (6) Openness * ~Conscientiousness * ~Extraversion * Agreeableness * Neuroticism * Gender * Age * ~Education; (7) Openness * Conscientiousness * Agreeableness * Neuroticism * ~Gender * ~Age * Education; (8) Openness * Conscientiousness * Extraversion * ~Agreeableness * ~Neuroticism * ~Gender * ~Age * Education; (9) Openness * Conscientiousness * Extraversion * Agreeableness * Neuroticism * ~Gender * Education.

## 4. Discussion

### 4.1. Personality Configurations and Media Vicarious Traumatization

This study explored the relationship between the configurations of the Big Five personality traits and media vicarious traumatization based on the report of the MU5735 airplane crash. Analyzing the online questionnaire data collected from 331 university students, this study found five configurations of personality traits that are more vulnerable to media vicarious traumatization, with each personality trait belonging to at least one configuration. According to Table 6, we obtain the following conclusion:

Solution 1 (Conscientiousness * Agreeableness * Neuroticism) indicates that university students who have high levels of conscientiousness, agreeableness, and neuroticism easily suffer from media vicarious traumatization. Zhang et al. [61] found that people who are high on agreeableness show more frequent information-seeking behavior when faced with uncertain situations, giving them more access to relevant information. Additionally, excessive information exposure is an important cause of media vicarious traumatization [14]. According to Nikčević et al.’s and Liu et al.’s studies, individuals who are high on conscientiousness tend to feel more stress, anxiety, and depression in emergencies, which can lead to psychological problems [62,63]. Moreover, a previous study argued that neuroticism is also a significant driver of vicarious trauma [19]. Therefore, media vicarious traumatization occurs more often in university students with the combined personality of high conscientiousness, agreeableness, and neuroticism.

Solution 2 (Conscientiousness * Extraversion * Agreeableness) denotes that university students with high conscientiousness, extraversion, and agreeableness have a higher risk of suffering media vicarious traumatization. Luengo et al. [64] identified that conscientiousness and agreeableness are critical predictors of prosocial behavior (i.e., voluntary acts such as sharing, giving, caring, solace, and assistance that are intended to support others [65]). Consequently, university students who have high levels of conscientiousness and agreeableness can better empathize with the pain and sorrow of others and provide care and comfort to those involved in air crashes. Moreover, university students high on extraversion tend to seek social interactions on social media [66]. When they frequently talk about accidents with others in this way, their negative emotions are further enhanced [3]. Therefore, under the combined effects of these three personality traits, university students are more easily influenced by the content of disaster coverage and thus become more susceptible to media vicarious traumatization.

Solution 3 (~Conscientiousness * Extraversion * ~Agreeableness * Neuroticism) shows that university students with high extraversion, neuroticism, as well as low conscientiousness and agreeableness, more easily experience media vicarious traumatization. Bowden-Green [66] found that people with high extraversion are usually better at searching for media information, meaning that university students who are high on this personality trait may be more excellent at finding detailed disaster coverage. Furthermore, individuals with high neuroticism and low conscientiousness are at higher risk of developing depressive symptoms [67]. Meanwhile, university students with both high neuroticism and low agreeableness are more likely to generate negative emotions such as pain [68]. As a result, university students with personality traits like solution 3 are more sensitive to disaster coverage, thus falling into a negative psychological state and becoming more vulnerable to media vicarious traumatization.

Solution 4 (Openness * Extraversion * Agreeableness * Neuroticism) shows that university students high on openness, extraversion, agreeableness, and neuroticism are prone to experience media vicarious traumatization. Previous studies have identified that all these four personality traits positively relate to media usage frequency [69,70]. Continuous and high exposure to disaster-related information is an essential trigger for individuals to experience media vicarious traumatization [14]. In addition, agreeableness is a personality trait that makes a person vulnerable [21]. In other words, individuals with this trait usually have greater empathy and are easily influenced to fall into sadness by external factors. Moreover, university students with high neuroticism tend to have low levels of self-efficacy and are inclined to internalize emotions [20]. When wrapped up in negative emotions such as anxiety, sadness, and worry, these students cannot self-regulate and channel them in time. The accumulation of emotions eventually leads to the emergence of vicarious trauma symptoms [19]. So accordingly, under the combined influence of high openness, extraversion, agreeableness, and neuroticism, university students are more susceptible to media vicarious traumatization.

Solution 5 (~Openness * Extraversion * Agreeableness * ~Neuroticism) denotes that university students with high extraversion, agreeableness, as well as low openness and neuroticism have a higher risk of suffering from media vicarious traumatization. Extraversion is a significant personality trait for an individual to be proactive in acquiring information [66,69,70]. Previous research has found that highly agreeable individuals experience stronger negative emotions when stimulated by information [71,72]. Furthermore, being sensitive to emotional stimuli leads to more agreeable people being more likely to predict higher sadness [73]. Therefore, university students are still vulnerable to media vicarious traumatization under the combined effect of high extraversion and high agreeableness, though their levels of openness and neuroticism are low.

For the raw coverage, solutions 1, 2, 3, 4, and 5 are 44.6%, 50.5%, 28.5%, 37.2%, and 33.9%, respectively. These five solutions represent sufficient configurations of causal conditions for media vicarious traumatization.

### 4.2. Personality Configurations (Including Sociodemographic Variables) and Media Vicarious Traumatization

The results suggest that differences in gender, age, and education among university students also influence their likelihood of experiencing media vicarious traumatization and form nine configurations under the interaction with personality traits. We can obtain the following conclusions according to Table 7: First, in terms of gender, six solutions (solutions 1–6) relate to females and only three solutions (solutions 7–9) relate to males, indicating that female university students are more likely to experience media vicarious traumatization than male university students. Second, by age, students aged 18–24 (solutions 1–4 and solutions 7–8) are at greater risk of experiencing media vicarious traumatization than students aged 25–29. Third, by education, undergraduate students (solutions 1–3 and solutions 5–6) are more vulnerable to media vicarious traumatization than graduate students (solutions 7–9). Overall, female undergraduate students and male graduate students are more likely to have traumatic symptoms after exposure to disaster reports. Furthermore, female university students with less education and younger age are at the greatest risk for trauma.

The interaction of sociodemographic variables and personality traits can also influence the development of media vicarious traumatization. In the group of female university students, high extraversion, high agreeableness and high neuroticism are important traits that leads to their traumatic experiences. While in the group of male university students, high openness and high conscientiousness are important traits that lead to their experience of trauma.

## 5. Implications and Future Research Direction

### 5.1. Implications of the Study

Our research has implications in the three dimensions listed below:

First, with the discussed results, this study extends the application of the Big Five theory. Although the function of personality traits has been discussed in a large number of studies on the use of media, few scholars have focused on how personality traits affect media vicarious traumatization, especially in university student populations. This study contributes to the enrichment of personality research by applying the Big Five theory to research media vicarious traumatization.

Second, given that individuals’ reactions and behavior result from the combined functions of various personality traits and the impossibility for a single trait to function alone, the combined effects of different personality traits should be fully considered in research. This study reveals the relationship between the combinations of personality traits and media vicarious traumatization by fsQCA and emphasizes the importance of the combined effects of personality traits on individuals, which promotes subsequent research on related topics.

Third, uncovering the relationship between personality traits and media vicarious traumatization, our research provides references for developing psychological interventions for trauma victims. In particular, (1) to identify vulnerable groups according to the sociodemographic characteristics and the results of personality trait tests of university students so that targeted interventions can be provided in time when crisis events occur; (2) targeted intervention strategies for psychological assistance can be developed based on personality configuration characteristics.

### 5.2. Limitations and Future Research Direction

This research has five limitations. First, the sample was limited to university students in the Chinese mainland, which may restrict the explanatory power of the findings. Future studies may consider broadening the sample by conducting surveys for different countries/regions, ages, and occupational groups to get a more generalized conclusion. Second, self-reporting was the only method used to gather data for this study, which may involve a response bias. For increased validity, researchers may try to collect objective data from psychological experiments or internet ethnography. Third, this study used a simplified version of the personality inventory, limiting the number of indicators. The full version may be used in the future to collect more complete data. Fourth, the duration and frequency of exposure were not assessed, which indicates that some respondents were at greater risk for vicarious trauma than others as a matter of the duration and frequency of exposure, which is a sort of selection bias. Fifth, another sort of bias that may be involved in this study is that data were collected online, indicating that participation was more related to those with smartphones or PC.

## 6. Conclusions

Our research explored the influential relationship between personality traits and media vicarious traumatization. According to the results, five combinations of personality traits easily cause media vicarious traumatization. In addition, this study highlights the influence of gender, age, and education when interacting with personality traits on the development of media vicarious traumatization. This study is necessary as media vicarious traumatization is becoming increasingly common. The findings of this study contribute to a deeper comprehension of the influential relationship between personality traits and vicarious trauma, enrich the literature in the field of research on media vicarious traumatization, help identify trauma victims, and provide guidance for the development of interventions.

## Figures and Tables

**Table 1:** Sociodemographic characteristics of participants (n = 331).

Items	Content	Frequency	Percentage
Gender	Male	186	56.2%
	Female	145	43.8%
Education	Undergraduate student	264	79.8%
	Graduate student	67	20.2%
Age (Mean = 22.63, SD = 2.67)	18–24	241	72.8%
	25–29	90	27.2%

Note: SD = Standard Deviation.

**Table 2:** Reliability and convergent validity (the Big Five Inventory-Short Version and Media Vicarious Traumatization Scale).

Variables	Criteria	Loadings
Openness (α = 0.79; CR = 0.87; AVE = 0.70)	I see myself as someone who is original, comes up with new ideas.	0.80
	I see myself as someone who values artistic, aesthetic experiences.	0.82
	I see myself as someone who has an active imagination.	0.89
Conscientiousness (α = 0.72; CR = 0.84; AVE = 0.64)	I see myself as someone who does a thorough job.	0.74
	I see myself as someone who tends to be lazy.	0.79
	I see myself as someone who does things efficiently.	0.85
Extraversion (α = 0.76; CR = 0.85; AVE = 0.66)	I see myself as someone who is talkative.	0.79
	I see myself as someone who is outgoing, sociable.	0.87
	I see myself as someone who is reserved.	0.78
Agreeableness (α = 0.76; CR = 0.86; AVE = 0.67)	I see myself as someone who is sometimes rude to others.	0.82
	I see myself as someone who has a forgiving nature.	0.86
	I see myself as someone who is considerate and kind to almost everyone.	0.78
Neuroticism (α = 0.74; CR = 0.84; AVE = 0.64)	I see myself as someone who worries a lot.	0.74
	I see myself as someone who gets nervous easily.	0.88
	I see myself as someone who remains calm in tense situations.	0.78
Media vicarious traumatization (α = 0.87; CR = 0.91; AVE = 0.61)	I was exposed to distressing news and experiences via media.	0.77
	I find myself distressed by reading the stories and situations on media.	0.61
	It is hard to stay positive and optimistic given some of the information I get from the media.	0.83
	I find myself thinking about distressing news on media.	0.72
	Sometimes I feel helpless because I cannot give help to people in need.	0.75
	Sometimes I feel overwhelmed by reading the media reports.	0.80
	I find it difficult to deal with the media content.	0.77

**Table 4:** Analysis of necessary conditions.

Conditions Tested	Consistency	Coverage
Extraversion	0.693	0.776
~Extraversion	0.670	0.658
Agreeableness	0.727	0.758
~Agreeableness	0.614	0.645
Conscientiousness	0.732	0.720
~Conscientiousness	0.578	0.646
Neuroticism	0.665	0.709
~Neuroticism	0.621	0.638
Openness	0.728	0.725
~Openness	0.634	0.698
Gender	0.481	0.574
~Gender	0.519	0.483
Age	0.261	0.501
~Age	0.740	0.531
Education	0.242	0.624
~Education	0.758	0.497

Note: ~ denotes the negation of a variable.

**Table 5:** Truth table (partial).

E	A	C	N	O	Number	MVT	Raw Consist.	PRI Consist.	SYM Consist.
1	1	1	1	1	42	1	0.913014	0.748091	0.749746
1	1	0	1	1	5	1	0.938887	0.67082	0.670821
1	1	1	1	0	5	1	0.92592	0.662302	0.664041
0	1	1	1	0	13	1	0.916728	0.646601	0.655346
1	0	0	1	0	4	1	0.918989	0.640696	0.640696
1	1	0	0	0	5	1	0.914211	0.625409	0.625408
1	0	0	1	1	9	1	0.914398	0.624197	0.636066
0	1	1	1	1	20	1	0.90026	0.621359	0.622954
1	1	1	0	0	10	1	0.901145	0.609952	0.617772
1	1	1	0	1	38	1	0.876065	0.600097	0.619835
1	0	1	1	0	4	0	0.924862	0.598252	0.598252
1	0	1	1	1	10	0	0.918067	0.596792	0.603014
1	1	0	0	1	9	0	0.895955	0.568846	0.568845
0	1	0	1	0	14	0	0.889126	0.553847	0.569881
1	0	1	0	1	12	0	0.90572	0.547957	0.547958
1	0	1	0	0	4	0	0.905534	0.547702	0.547702

Notes: E = Extraversion; A = Agreeableness; C = Conscientiousness; N = Neuroticism; O = Openness; MVT = Media Vicarious Traumatization.

**Table 6:** Analysis of sufficient conditions (personality traits).

Conditions	Solutions				
	1	2	3	4	5
Openness				●	Ⓧ
Conscientiousness	●	●	Ⓧ		
Extraversion		●	●	●	●
Agreeableness	●	●	Ⓧ	●	●
Neuroticism	●		●	●	Ⓧ
Consistency	0.874	0.853	0.903	0.908	0.891
Raw coverage	0.446	0.505	0.285	0.372	0.339
Unique coverage	0.075	0.054	0.058	0.005	0.018
Solution coverage	0.683				
Solution consistency	0.828				

Notes: Black circles (●) indicate the presence of a condition, and white crossed-out circles (Ⓧ) indicate the absence of a condition. Blank spaces indicate that the condition does not contribute to the configuration.

**Table 7:** Analysis of sufficient conditions (sociodemographic variables and personality traits).

Conditions	Solutions								
	1	2	3	4	5	6	7	8	9
Openness			●	●	Ⓧ	●	●	●	●
Conscientiousness	Ⓧ	●		●	●	Ⓧ	●	●	●
Extraversion	●	●	●	●	Ⓧ	Ⓧ		●	●
Agreeableness	Ⓧ	●	Ⓧ	●	●	●	●	Ⓧ	●
Neuroticism	●	●		Ⓧ	●	●	●	Ⓧ	●
Gender	●	●	●	●	●	●	Ⓧ	Ⓧ	Ⓧ
Age	Ⓧ	Ⓧ	Ⓧ	Ⓧ	●	●	Ⓧ	Ⓧ	
Education	Ⓧ	Ⓧ	Ⓧ		Ⓧ	Ⓧ	●	●	●
Consistency	0.915	0.985	0.947	0.962	0.948	0.946	0.929	0.970	0.922
Raw coverage	0.092	0.089	0.112	0.100	0.050	0.040	0.073	0.030	0.086
Unique coverage	0.005	0.010	0.013	0.028	0.021	0.011	0.015	0.012	0.028
Solution coverage	0.345								
Solution consistency	0.927								

Notes: Black circles (●) indicate the presence of a condition, and white crossed-out circles (Ⓧ) indicate the absence of a condition. Blank spaces indicate that the condition does not contribute to the configuration.

## Data Availability

Data are available upon special request from the corresponding author.
